# Screening for Antibiotic Resistance Genes in Bacteria and the Presence of Heavy Metals in the Upstream and Downstream Areas of the Wadi Hanifah Valley in Riyadh, Saudi Arabia

**DOI:** 10.3390/antibiotics13050426

**Published:** 2024-05-08

**Authors:** Norah M. Al-Otaibi, Bassam Alsulaiman, Fahad M. Alreshoodi, Lenah E. Mukhtar, Sulaiman M. Alajel, Norah M. Binsaeedan, Fahad M. Alshabrmi

**Affiliations:** 1 Executive Department of Reference Laboratories, Research and Laboratories, Saudi Food and Drug Authority (SFDA), Riyadh 13513, Saudi Arabia; nmaotaibi@sfda.gov.sa (N.M.A.-O.); bassamalsuliman@gmail.com (B.A.); smotibi@sfda.gov.sa (S.M.A.); nmsaeedan@sfda.gov.sa (N.M.B.); 2 Department of Medical Laboratories, College of Applied Medical Sciences, Qassim University, Buraydah 51452, Saudi Arabia

**Keywords:** antibiotic resistance, AMR, heavy metals, ARG, water, pollution, Wadi Hanifah, Riyadh, Saudi Arabia

## Abstract

Valley surface water is considered a focal public health concern owing to the presence of multi-drug-resistant bacteria. The distribution of antimicrobial resistance (AMR) bacteria in the surface water is affected by the presence of multiple factors, including antibiotics coming from wastewater discharge or other contaminant sources such as pharmaceuticals, biocides, and heavy metals. Furthermore, there is evidence suggesting that high levels of antibiotic resistance genes (ARGs) can be transferred within bacterial communities under the influence of heavy metal stress. Hence, the primary aim of this study is to investigate the presence of heavy metals and bacterial ARGs in upstream as well as downstream locations of Wadi Hanifah Valley in Riyadh, Saudi Arabia. Sample collection was conducted at eighteen surface water sites within the valley in total. The selection of ARGs was associated with the most common antibiotics, including β-lactam, tetracycline, erythromycin, gentamicin, sulphonamide, chloramphenicol, vancomycin, trimethoprim, and colistin antibiotics, which were detected qualitatively using polymerase chain reaction (PCR) technology. The tested antibiotic resistance genes (ARGs) included (*bla_NDM-1_* (for the antibiotic class Beta-lactamases), *mecA* (methicillin-resistant *Staphylococcus aureus*), *tet(M)* and *tet(B)* (for the antibiotic class Tetracycline), *ampC* (for the antibiotic class Beta-lactamases), *vanA* (for the antibiotic class vancomycin), *mcr-1* (for the antibiotic class colistin), *erm(B)* (for the antibiotic class erythromycin), *aac6′-Ie-aph2-Ia* (for the antibiotic class Gentamicin), *sulII* (for the antibiotic class sulphonamide), *catII* (for the antibiotic class Chlorophincol), and *dfrA1* (for the antibiotic class trimethoprim). Moreover, an assessment of the levels of heavy metals such as lithium (Li), beryllium (Be), chromium (Cr), cobalt (Co), arsenic (As), cadmium (Cd), tin (Sn), mercury (Hg), and lead (Pb) was conducted by using inductively coupled plasma mass spectrometry (ICPMS). According to our findings, the concentrations of sulphonamide, erythromycin, and chloramphenicol ARGs (*erm(B)*, *sulII*, and *catII*) were observed to be the most elevated. Conversely, two ARGs, namely *mecA* and *mcr-1*, were not detected in the samples. Moreover, our data illustrated a significant rise in ARGs in the bacteria of water samples from the upstream sites as compared with the water samples from the downstream sites of Wadi Hanifah Valley. The mean concentration of Li, Be, Cr, Co, As, Cd, Sn, Hg, and Pb in the water samples was estimated to be 37.25 µg/L, 0.02 µg/L, 0.56 µg/L,0.32 µg/L, 0.93 µg/L, 0.01 µg/L, 200.4 µg/L, 0.027 µg/L, and 0.26 µg/L, respectively, for the selected 18 sites. Furthermore, it was revealed that the concentrations of the screened heavy metals in the water samples collected from various sites did not surpass the maximum limits set by the World Health Organization (WHO). In conclusion, this study offers a concise overview of the presence of heavy metals and ARGs in water samples obtained from the Wadi Hanifah Valley in Riyadh, KSA. Such findings will contribute to the ongoing monitoring and future risk assessment of ARGs spread in surface water.

## 1. Introduction

Antimicrobial resistance (AMR) remains a major universal challenge for the global community [1]. It has been reported that more than 1.27 million deaths in 2019 have been reported to be linked to AMR, and the numbers are expected to rise in the coming years without unified global action plans [2,3]. The usage of antimicrobials in animal food manufacture is almost three times more than that of human food [4]. This is because antibiotics can be used for non-therapeutic purposes, such as growth promotors in animals, which is an important factor in the emergence of antibiotic resistance [5]. The worldwide usage of antibiotics for human purposes has surged by 65% from 2000 to 2015, primarily attributed to their widespread availability and utilization, especially in low- and middle-income nations [6]. Furthermore, by 2030, antibiotic consumption is expected to cause an increase in animals by 200,235 tons, and an increase to 13,600 tons in the breeding of aquatic animals [7]. Antimicrobial resistance also poses a significant threat to the global economy, with an estimation of a reduction in global GDP by USD 100–210 trillion. In addition, according to the Centers for Disease Control and Prevention (CDC), the United States incurs an annual financial burden of 55 billion USD due to antimicrobial resistance, with 20 billion USD associated with healthcare costs and a further 35 USD attributed to reduced productivity [8].

The global significance of environmental waters serving as reservoirs for antimicrobial resistance bacteria (ARB) and antibiotic resistance genes (ARGs) is well acknowledged as a public health concern [9]. Currently, wastewater treatment plants (WWTPs) predominantly release their effluent into rivers, valleys, and lakes [10]. Surface water is considered an ecosystem for marine animals and a source of drinking water for livestock, and plays a vital role in the irrigation system [11]. It has been reported that WWTPs harbor microbes, antibiotic residues, disinfectants, ARB, and ARGs that mainly originate from hospital wastes, pharmaceutical manufacturing wastes, runoff from aquaculture lands, plant-based food productions, and livestock [12,13]. 

Furthermore, antimicrobial agents are not completely metabolized after human and animal infections, so they end up being excreted as an active substance into sewage water [14]. Thus, the antimicrobials present in the water system can lead to microorganisms adapting to and surviving the currently used antibiotics and acquiring new variance factors and novel resistance pathways [3,15]. More recently, a high concentration of antibiotic residues has been recorded previously from the samples recovered from surface water [16,17]. 

The dissemination of AMR is not solely affected by the antibiotics; there are other additional factors, including pharmaceuticals, pesticides, and heavy metals in water, which can induce selective pressure stress in bacteria, contributing to the selection of antimicrobial-resistant bacteria [18]. Research indicates that high levels of ARGs can be transferred under heavy metal stress in bacterial communities within sludge [19]. 

Heavy metals induce bacterial resistance through both direct and indirect pathways. In the direct pathway mechanisms, heavy metals trigger antimicrobial resistance via co-resistance and cross-resistance mechanism [20]. Co-selection involves different resistance determinants occurring on the same genetic element, such as a plasmid. Cross-resistance occurs when a similar genetic factor is responsible for resistance to both antibiotics and heavy metals [20]. The indirect pathway is activated when exposure to antibiotics and metals stimulates biofilm formation in microbial populations, allowing bacteria to persist in the presence of antimicrobial compounds [21]. 

Thus, considering the crucial role of environmental water in the expansion of antimicrobial drug resistance, in our study, water samples from Wadi Hanifah were assessed for the presence of heavy metals and ARGs. Wadi Hanifah is a critical surface water source located in Saudi Arabia’s capital city (Riyadh), and it covers an area of about 4400 km^2^ from the northwest to the southeast of Riyadh. The length of Wadi Hanifah is about 120 km. However, to date, there is no published report investigating the presence of ARGs within Wadi Hanifah and its environmental pollution, such as the heavy metals in aquatic ecosystems. Therefore, it is essential to screen ARGs and heavy metals in Wadi Hanifah and understand the potential dissemination of ARGs in water systems. Thus, this study aims to employ culture-independent methods to characterize and compare the frequency of antibiotic resistance genes between the upstream and downstream sections of Wadi Hanifah Valley. Additionally, the study’s objective is to examine the concentrations of heavy metals throughout the water system. This study provides a preliminary report of the heavy metal and ARG prevalence in water samples from Wadi Hanifah, and it will aid in controlling the expansion of these components in surface water. 

## 2. Materials and Methods

### 2.1. Sample Collection

**Figure 1:**
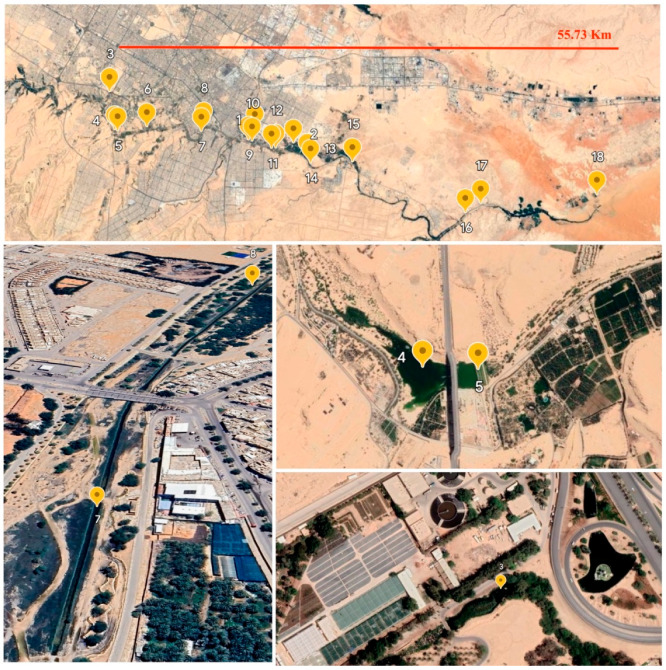
Google Earth map that highlights the 18 sites of collected water samples from both upstream and downstream areas in Wadi Hanifah Riyadh, Kingdom of Saudi Arabia (upstream area denotes sites # 1–12 and downstream area denotes sites # 13–18). In addition, this figure shows several sites around the valley and their surrounding areas, including WWTP stations, farms, and manufacturers.

Water samples were obtained in duplicate from 18 distinct sites in Wadi Hanifah, Riyadh, KSA, in September 2022 (Figure 1). These samples were carefully preserved in sterile one-liter glass vials. Subsequently, the samples were kept on ice and promptly transported to the laboratory for immediate processing through direct filtration. One liter of the collected sample was filtered through 0.45 micro-meter-pore-size membrane filters (Millipore, Bedford, MA, USA), and the resulting filters were placed in glass bead tubes for DNA extraction experiments. Simultaneously, the pH and temperature of the samples were directly measured and analyzed. 

### 2.2. Extraction of Genomic DNA

Genomic DNA was extracted from the filtered water samples using the Power Water kit (Qiagen, Hilden, Germany) following the manufacturer’s recommendations. Subsequently, the concentration and purification of the extracted DNA were evaluated using a QIAxpert spectrophotometer (Qiagen, Hilden, Germany).

### 2.3. Detection of Antibiotic-Resistant Genes (ARGs) 

Following DNA extraction, 12 ARGs, which represent 10 antibiotic classes, were detected using polymerase chain reaction (PCR) technology. A thermal cycler (Bio-Rad, Hercules, CA, USA) was used to detect the ARGs genes (*bla_NDM-1_* (for the antibiotic class Beta-lactamases), *mecA* (methicillin-resistant *Staphylococcus aureus*), *tet*(*M*) and *tet*(*B*) (for the antibiotic class Tetracycline), *ampC* (for the antibiotic class Beta-lactamases), *vanA* (for the antibiotic class vancomycin), *mcr-1* (for the antibiotic class colistin), *erm*(*B*) (for the antibiotic class erythromycin), *aac6′-Ie-aph2-Ia* (for the antibiotic class Gentamicin), *sulII* (for the antibiotic class sulphonamide), *catII* (for the antibiotic class Chlorophincol), and *dfrA1* (for the antibiotic class trimethoprim)). Table 1 shows the list of the primers used in the current study. All the primers used in this study were pre-made in lyophilized form and optimized for use with 5x FIREPol^®^ master mixes (Macrogen, Seoul, Republic of Korea). A master 100× stock was prepared in water for each primer and was diluted to a working stock of 10×. The setups for the reactions were arranged in a total volume of 20 µL per reaction. The extracted DNA from water samples was added to a reaction mixture that also contained 5x FIREPol^®^ master mix and 0.2 μM of each primer. Table 2 summarizes the PCR cycling conditions for each primer used. Negative controls that contained all reaction components except the DNA sample were used to identify PCR contamination. The PCR products underwent electrophoresis at 80 volts for 60 min on 1.5% agarose gel (Bio-Rad, USA), and the gel documentation system (Bio-Rad, USA) was utilized to capture images.

### 2.4. Determination of Heavy Metals

The analysis of heavy metals in water samples was conducted utilizing the 7900ICP-MS model from Agilent, Santa Clara, CA, USA, employing inductively coupled plasma mass spectrometry (ICP-MS). The reflected power for ICP was set at 1500 W, with a plasma gas flow of 15 L/min and an auxiliary (makeup) gas flow of 1 L/min. Prior to sample injection, specifications for sensitivity, oxide ratio, and doubly charged ratio were carefully examined to minimize interference.

### 2.5. Quality Control

Ensuring the reliability and accuracy of the results, a certified reference material was created and analyzed with each batch of analysis. The accepted recovery range was established to be between 70% and 120%, and the relative standard deviations (RSDs) for repeated measurements were set at <20%. Quality control techniques included the use of solvent blanks, fresh calibration standards, and spiked samples. The minimum correlation coefficient (r^2^) required for the heavy metals was set at >0.995 (Table 3).

### 2.6. Statistical Analysis

Prism version 9.1.1 was employed for data analysis and visualization. The results were presented as mean ± SE. Subsequently, the T-test was utilized to examine significant differences between upstream and downstream locations concerning antibiotic resistance genes, ARGs, and heavy metals. Additionally, the Pearson correlation coefficient (r) was applied to assess the correlation between ARGs and heavy metals in the data.

## 3. Results

**Figure 2:**
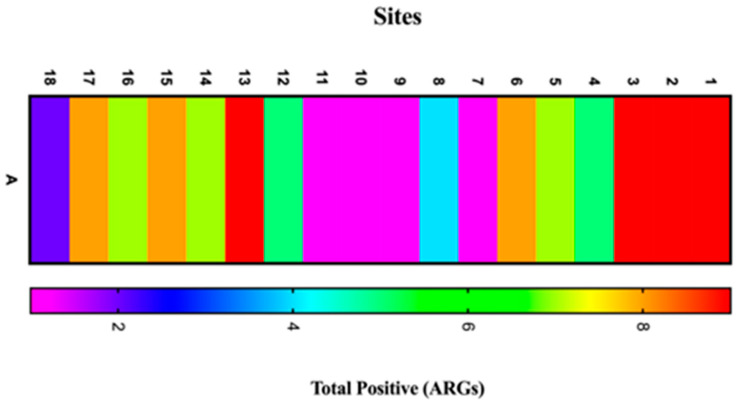
A heat map representing the total frequency of positive ARGs in the targeted locations, as shown in the first three locations containing a high number of ARGs.

Here, 12 ARGs were screened in a total of 18 sites. In brief, the results from our screening analysis revealed that ten of the ARGs (*bla_NDM-1_*; *tet*(*M*); *ampC*; *vanA*; *erm*(*B*); *aac* (*6′*)*-Ie-aph* (*2″*)*-Ia*; *sulII*; *dfrA1*; and *catII*) were identified via PCR. However, two ARGs (*mec* and *mcr-1*) were not detected in all sites along Wadi Hanifah Valley, Riyadh, Kingdom of Saudi Arabia. In fact, three locations in the upstream sites out of 18 were found to harbor a high level of ARGs (Figure 2).

**Figure 3:**
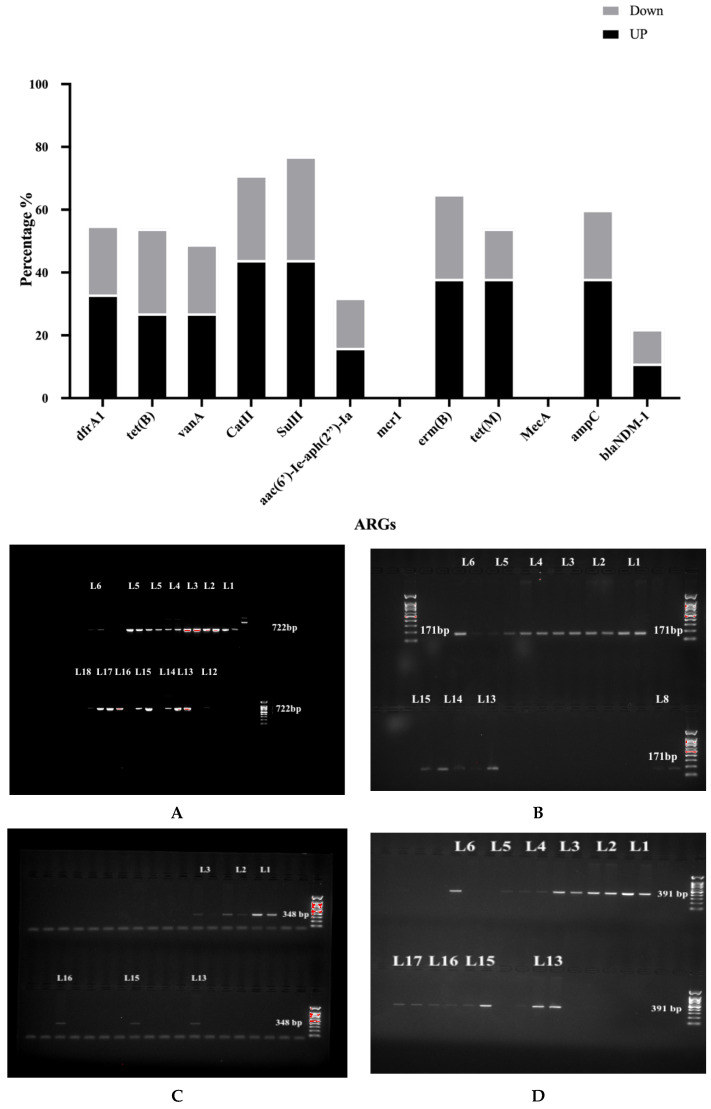
A bar graph and gel pictures highlighting the frequency of antibiotic resistance genes in different sites in the Wadi Hanifah, Riyadh, Kingdom of Saudi Arabia. Gel pictures illustrate the amplification of four important genes: (**A**) the *sulII* gene was found in 14 locations out of 18, (**B**) the *tet(M)* gene was detected in 7 locations from the upstream area and three locations in the downstream area, (**C**) *aac*(*6′*)*-le-aph*(*2″*) was positive in 6 locations, and (**D**) *dfrA1* was positive in 1 locations. (L denotes location. Samples were run on the gel in duplicate).

As shown in Figure 3, only 22.2% of all samples were contaminated with the multi-resistance beta-lactam antibiotics gene (*bla_NDM-1_*). Our data indicated that all samples were negative for the *bla_NDM-1_* gene except site # 5; 12 were negative from the upstream areas and # 13 and 14 sites located in the downstream part. The *ampC* gene was detected in 11 out of 18 sites (61.1): sites # 1–6, 8, and 12 from the upstream area, and sites # 13–16 and 17 from the downstream area. As illustrated in Figure 3, our data showed that water samples collected from the upstream area (sites # 1–6, and 8) were positive for *the tet*(*M*) gene. This gene was also amplified in samples extracted from the downstream sites (# 13, 14, and 15). The gene *tet*(*B*) was observed in both upstream and downstream sites (# 1, 2, 3, 6, and 12–17). The percentage of both *tet*(*M*) and *tet*(*B*) genes from total sites was 55.5% (Figure 3).

The gene *erm*(*B*) was revealed at a frequency of 66.7% in Wadi Hanifah Valley, from the samples that were collected from sites # 1 to 6 and 7 from the upstream areas and sites # 13 to 17 from the downstream areas (Figure 3). 

The *aac*(*6′*)*-Ie-aph*(*2″*)*-Ia* gene was detected in six sites of Wadi Hanifah Valley, three from upstream sites (# 1–3) and three from downstream sites (#13, 15 and 17). The overall frequency of antibiotic resistance gene *aac*(*6′*)*-Ie-aph*(*2″*)*-Ia* was 33.3% in Wadi Hanifah (Figure 3). The *sulII* gene was detected in 44.4% of samples collected from upstream sites (# 1–6, 8, and 12) and in 33.3% of water samples from downstream areas (sites # 13–18). The results in Figure 3 indicated that 77.7% of both sites were contaminated with sulphonamide antibiotics. Our data showed that 72.2% of the Wadi Hanifah sites were contaminated with the *catII* gene. The *catII* gene was found in seven samples collected from sites # 1, 3–6, and 9–10 from upstream points and sites # 12–18 from downstream points. These data showed that the Wadi Hanifah Valley was also contaminated with chloramphenicol (Figure 3). 

The *vanA* gene was observed in water samples taken from upstream sites # 1–3, 7, and 11, and in sites # 13–14, 16, and 17 collected from the downstream areas, suggesting that these areas (50% from all samples) were contaminated with vancomycin antibiotics (Figure 3). 

Finally, our data indicated that water samples collected from the upstream sites (sites # 1–6) and the downstream sites (sites # 13, 15, 16, and 17) were positive for *the dfrA1* gene with a frequency of 55.5%. Thus, these areas were contaminated with trimethoprim antibiotics. Interestingly, none of our samples contained *mcr-1* or *mecA* genes (Figure 3). 

In summary, our data showed that ARGs levels increased significantly in upstream locations as compared with the downstream locations (*p* = 0.002). 

### 3.1. Heavy Metal Contents in Water Samples

**Figure 4:**
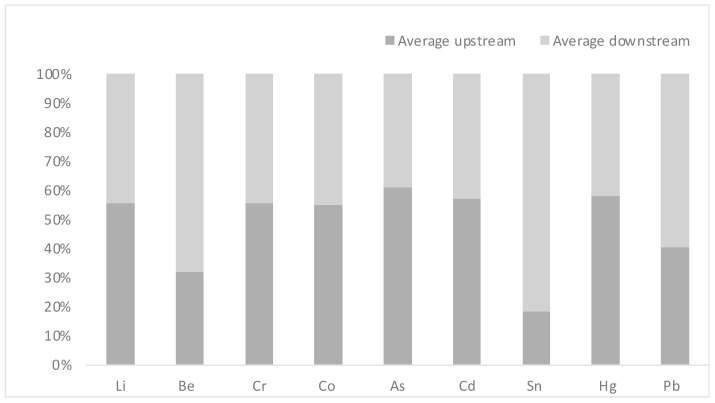
Percentage of heavy metal concentration determined by using ICP-MS for 18 water samples in both the upstream and downstream sites.

The average concentration of Li, Be, Cr, Co, As, Cd, Sn, Hg, and Pb were calculated and determined by using ICP-MS (Table 4). The following is the order in which the concentration of metal(loid)s in water samples was expressed as a mean concentration: Sn, Li, As, Cr, Co, Pb, Hg, Be, and Cd for 65.86 µg/L, 40.40 µg/L, 1.08, 0.61,0.34, 0.20, 0.03, 0.013, and 0.011 µg/L in the upstream areas, respectively, and Sn 296.527 µg/L, Li 32.32 µg/L, As 0.690 µg/L, Cr 0.489 µg/L, Pb 0.300 µg/L, Co 0.281 µg/L, Be 0.027 µg/L, Hg 0.025 µg/L, and Cd 0.008 µg/L in the downstream areas. The percent of total metal(loid)s in water samples from both upstream and downstream sites is revealed in Figure 4.

### 3.2. Correlation between ARGs and Heavy Metals

The Pearson correlation coefficient (r) test illustrated that heavy metals exhibited a correlation but one that was non-significant in terms of the ARGs (R = 0.066, 95% confident interference = −0.41 to 0.5; R square = 0.004).

## 4. Discussion

Heavy metals in aquatic systems may interfere with ARGs by influencing co-selection and cross-resistance mechanisms in bacteria, even at low levels of exposure [32,33,34]. Recently, few studies reported environmental health concerns from ARGs due to heavy metal interaction with drug-resistant bacteria [4]. Surface water is an emerging field for determining the origin of AMR in terms of one health approach [35]. Therefore, there is a massive need for a primary assessment of surface-water heavy metal concentrations and ARG prevalence in Saudi Arabia., although, the Wadi Hanifah Valley constitutes a strategic part of Riyadh city and a significant natural landmark of the region. This is the first study that targets the upstream and downstream sites of the valley to assess the concentration of heavy metals and the contamination of ARGs in the water body. Thus, monitoring environmental risk is a crucial step in controlling AMR.

Our results in general show that the upstream area of Wadi Hanifah Valley has a higher level of ARGs. In this study, the significantly higher ARG percentage in the upstream area was mostly due to the presence of WWTPs in these locations. Surface water frequently receives effluents from WWTPs, which may contain antibiotic residues and antibiotic-resistant bacteria. As a result, surface water was a potential source for the dissemination of ARGs [36]. It has been observed that the discharge of WWTPs at upstream points caused an accumulation of ARGs in these areas. This finding agrees with the study by [16] that showed that the abundance of 23 ARGs in the upstream area of surface water samples taken from 38 sites in the Beijiang River, China, was 8.7 × 10^6^ copies/ng of DNA, and that in downstream areas was 3.17 × 10^6^ copies/ng of DNA [16]. On the contrary, WWTP effluents were not always contributors to the contamination of ARGs in the surface water bodies. To avoid the direct release of ARGs into the environment, WWTPs are required to be well designed and have efficient ARG removal mechanisms [37]. 

Moreover, it was observed that the concentrations of erythromycin, sulphonamide, and chloramphenicol ARGs (*erm*(*B*), *sulII*, and *catII*) were the most elevated in all the examined samples, while two ARGs (*mecA* and *mcr-1*) were not identified. Notably, in a 2016 study conducted in the Tordera River Basin in Catalonia, Spain, a noteworthy rise in the abundance of *ermB* and *sulI* genes was identified in biofilms obtained from locations downstream of the WWTP discharge points [38]. Furthermore, in Nigeria, 60 bacteria isolates in total from wastewater were tested against 41 ARGs, and the higher-prevalence ARG was *catA1* (58.3%), which encoded chloramphenicol resistance [39]. These results confirmed the significant risk of WWTP discharge into the environment promoting the spread of ARGs. There are several possible reasons for the high prevalence of erythromycin, sulphonamide, and chloramphenicol resistance, including the persistence of the antibiotics within the environment and the restrictions to eliminating and breaking them down, which in turn increases resistance in the pathogens [36]. The excessive consumption of the aforementioned antibiotics in farms and hospitals surrounding the valley may be a significant cause for the accumulation of these ARGs [40]. Therefore, additional investigation is needed to ascertain the reasons for the elevated occurrence of the *erm*(*B*), *sulII*, and *catII* genes. Investigating the sources of ABR’s increase will provide significant new data, including an understanding of resistance mechanisms that will be helpful in the prediction of AMR through the use of several technologies, including artificial intelligence [41]. In addition, these investigations will provide knowledge about ineffective antibiotics, which will help to mitigate the economic and health consequences associated with consuming inefficient antibiotics leading to bacterial resistance evolution [13].

The low prevalence of *mcr1* and *mecA*, which encoded both colistin and methicillin resistance, were expected in surface water due to the reduced use of methicillin and colistin. In the mid-1990s, colistin re-emerged as a last-line treatment against multiple-drug-resistant (MDR) Gram-negative pathogens [42]. Therefore, the presence of colistin is very limited in the environment. On the other hand, methicillin resistance only happens in certain bacteria within chromosomes (e.g., *Staphylococcus aureus*) that are found in freshwater at a low prevalence [43]. The European Medicines Agency (EMA) classifies colistin as an antibiotic that is extremely important for use in human medicine. This indicates that, to minimize the risk to the public’s health, its use in veterinary care should be restricted. Therefore, colistin prevalence in the environment should be assayed and measured to control its spread [44]. 

Overall, our findings indicate that the water samples under investigation remain within the permissible limits for surface waters according to the standards set by the WHO [45,46] and USEPA [47,48]. The results pertaining to metals such as Li, Be, Cr, As, Co, Cd, Su, Pb, and Hg offer valuable insights into the water quality of Wadi Hanifah Valley in Riyadh, Kingdom of Saudi Arabia. Furthermore, our data reveal no significant difference in heavy metal levels between the upstream and downstream sites. This aligns with the work conducted by [49], in which their findings showed extremely low levels of heavy metal pollution in the surface water of Heihe River [49]. Despite the low levels observed, its worth noting that even minimal exposure to heavy metals, such as Cu, Ag, Cr, and Zn, in environmentally relevant concentrations below the minimum inhibitory concentrations (MICs), has been reported to facilitate the conjugative transfer of antibiotic resistance genes between *E. coli* strains [34]. 

According to our data, the heavy metal concentrations and the ARGs are correlated with each other. However, the correlation was not found to be significant. Furthermore, our data indicate the possibility of increased metal pressure on pathogenic bacteria over time, resulting in the evolution of resistance mechanisms. Evaluating the risks associated with chemical and microbial pollutants in the environment is a crucial stage for identifying outbreak sources, and developing strategies and regulations to mitigate the risk of AMR. Heavy metals, antibiotics, and ARGs were measured in manures and soil samples taken from various feedlots in Shanghai [50]. ARGs and antibiotics displayed a weakly positive correlation [50]. On the contrary, in three urban lakes in Nanjing, China, Spearman analysis revealed a significant positive association between cadmium, lead, copper, *sul2*, and *strB* [51]. 

As commonly understood, improper environmental practices lead to the accumulation of pathogenic microbes and diverse chemical pollutants in the environment, thereby amplifying resistance mechanisms in bacteria. Hence, this study evaluated both heavy metals and antibiotic resistance genes to gauge the quality of surface water from a One Health perspective. The objective is to bolster health and economic policies, mitigating the spread and potential risks associated with bacterial resistance to antibiotics.

## 5. Conclusions

This study examined the presence of resistance genes related to commonly used antibiotics in bacteria in various water bodies, alongside the assessment of heavy metal concentrations. The proliferation of antimicrobial resistance, AMR, in surface water is not solely instigated by the presence of antibiotics originating from wastewater discharge, industrial and hospital waste, agricultural practices, and animal sources, but is also influenced by the existence of other factors such as heavy metals. The evaluation of AMR in the environment is a crucial step within the framework of a One Health approach, especially considering the irresponsible and excessive use of antimicrobials in agriculture, livestock, and human medicine. Inadequate antibiotic management, ineffective infection control, agricultural residue, environmental pollutants, and the movement of people and animals carrying resistant bacteria collectively contribute to the dissemination of resistance. Hence, this study delves into both heavy metal and antibiotic resistance genes to establish a foundational understanding of the content in the Wadi Hanifah Valley, thereby advancing our ability to manage AMR. It also underscores the urgency of implementing effective policies and technologies to control the contamination of the environment with ARGs.

## Figures and Tables

**Table 1:** A table listing the primers used in this study.

Gene	Oligo Name	Oligo Sequence (5′ to 3′)	References
*bla_NDM-1_*	NDM1-F	GGTTTGGCGATCTGGTTTTC	[22]
	NDM1-R	CGGAATGGCTCATCACGATC	
*mecA*	mecA F 1282	AAAATCGATGGTAAAGGTTGGC	[23]
	mecA R 1793	AGTTCTGCAGTACCGGATTTGC	
*ampC*	ampC-F	TTCTATCAAMACTGGCARCC	[24]
	ampC-R	CCYTTTTATGTACCCAYGA	
*tet*(*M*)	tet(M)-F	ACAGAAAGCTTATTATATAAC	[25]
	tet(M)-R	TGGCGTGTCTATGATGTTCAC	
*erm*(*B*)	ermB-F	CATTTAACGACGAAACTGGC	[26]
	ermB-R	GGAACATCTGTGGTATGGCG	
*aac*(*6′*)*-Ie-aph*(*2″*)*-Ia*	aac6-aph2F	CAGAGCCTTGGGAAGATGAAG	[27]
	aac6-aph2R	CCTCGTGTAATTCATGTTCTGGC	
*sulII*	Sul2-F	CGGCATCGTCAACATAACC	[28]
	Sul2-R	GTGTGCGGATGAAGTCAG	
*catII*	CatII-F	CCTGGAACCGCAGAGAAC	[29]
	CatII-R	CCTGCTGAAACTTTGCCA	
*vanA*	VanAF	GGGAAAACGACAATTGC	[30]
	VanAR	GTACAATGCGGCCGTTA	
*tet*(*B*)	tet(B)-F	CCTTATCATGCCAGTCTTGC	[28]
	tet(B)-R	GGAACATCTGTGGTATGGCG	
*dfrA1*	dhfrI-F	AAGAATGGAGTTATCGGGAATG	[28]
	dhfrI-R	GGGTAAAAACTGGCCTAAAATTG	
*mcr-1*	CLR5-F	ATCCCATCGCGGACAATCTC	[31]

**Table 2:** A table listing the antimicrobial resistance genes, positive control, and amplicon.

Gene	Positive Control	Amplicon Size (bp)
*blaNDM-1*	*Klebsiella pneumoniae* (ATCC 35657)	621
*mecA*	*Staphylococcus aureus* (ATCC 43300)	533
*ampC*	*Staphylococcus aureus* (Food isolate)	550
*tet*(*M*)	*Staphylococcus aureus* (ATCC 6538)	171
*erm*(*B*)	*Staphylococcus aureus* (ATCC 25923)	405
*aac*(*6′*)*-Ie-aph*(*2″*)*-Ia*	*Staphylococcus aureus* (Food isolate)	348
*sulII*	*Salmonella* (Food isolates)	722
*catII*	*Enterobacter. colacae* (ATCC 49141)	495
*vanA*	*Enterobacter* (Clinical isolates)	723
*tet*(*B*)	*Staphylococcus aureus* (ATCC 25923)	774
*dfrA1*	*E. coli* (ATCC 25922)	391
*mcr-1*	*Salmonella* (Food isolates)	177

**Table 3:** List of detected metals with percentage of recovery and method performance for the analysis of metals using ICP-MS.

Metals	Molecular Weight (g/mol)	Spik Con. (µg/L, n = 6)	Recovery (%)	Limit of Quantification (µg/L)	Uncertainty (%)
Lithium	6.941	5	88–111	13.4	19.8
Beryllium	9.012	60	101–104	1.2	6.6
Chromium	51.99	15	85–110	13.1	37.2
Cobalt	28.010	5	88–99	1.3	14.6
Arsenic	74.9	5	99–106	1.4	12.6
Cadmium	112.4	5	84–88	1.4	28.8
Lead	207.2	1	105–108	1.0	14.8

**Table 4:** Range and average ± SD of heavy metal concentrations in water samples from both upstream and downstream areas (µg/L).

Heavy Metal	Average Concentration Upstream (±SD)	Average Concentration Downstream (±SD)
Li	40.40 (±10)	32.32 (±2)
Be	0.01 (±0.003)	0.03 (±0.01)
Cr	0.61 (±0.3)	0.49 (±0.2)
Co	0.34 (±0.06)	0.28 (±0.1)
As	1.08 (±0.46)	0.69 (±0.1)
Cd	0.01 (±0.006)	0.01 (±0.01)
Sn	65.86 (±1.1)	296.53 (±55)
Hg	0.03 (±0.01)	0.03 (±0.004)
Pb	0.20 (±0.03)	0.30 (±0.06)

## Data Availability

The raw data supporting the conclusions of this article will be made available by the authors on request.
